# Lassa Fever in Travelers from West Africa, 1969–2016

**DOI:** 10.3201/eid2502.180836

**Published:** 2019-02

**Authors:** Aaron Kofman, Mary J. Choi, Pierre E. Rollin

**Affiliations:** Centers for Disease Control and Prevention, Atlanta, Georgia, USA

**Keywords:** Lassa fever, travel medicine, zoonotic diseases, viruses, West Africa, zoonoses

## Abstract

Lassa virus is a rodentborne arenavirus responsible for human cases of Lassa fever, a viral hemorrhagic fever, in West Africa and in travelers arriving to non–Lassa-endemic countries from West Africa. We describe a retrospective review performed through literature search of clinical and epidemiologic characteristics of all imported Lassa fever cases worldwide during 1969–2016. Our findings demonstrate that approximately half of imported cases had distinctive clinical features (defined as fever and >1 of the following: pharyngitis, sore throat, tonsillitis, conjunctivitis, oropharyngeal ulcers, or proteinuria). Delays in clinical suspicion of this diagnosis were common. In addition, no secondary transmission of Lassa fever to contacts of patients with low-risk exposures occurred, and infection of high-risk contacts was rare. Future public health investigations of such cases should focus on timely recognition of distinctive clinical features, earlier treatment of patients, and targeted public health responses focused on high-risk contacts.

Originally discovered in 1969, Lassa fever is a rodentborne viral hemorrhagic fever endemic to West Africa and caused by Lassa virus (*1*). The clinical course of Lassa fever is either not recognized or mild in 80% of patients; however, ≈20% of patients might experience severe disease, including facial swelling, hepatic and renal abnormalities, pulmonary edema, and hemorrhage. Although overall case-fatality rates for patients with Lassa fever is ≈1%, rates among hospitalized case-patients are >15% (*2*). Intravenous administration of the antiviral drug ribavirin has become the standard of care for treatment of Lassa fever, but data on the efficacy of intravenous ribavirin are limited. The original study among Lassa fever patients in Sierra Leone found survival to be significantly higher (p = 0.0002) among those who obtained ribavirin within the first 6 days of illness (55%) compared with those who never received the drug (5%) (*3*). In the United States, intravenous ribavirin use is still considered investigational and may only be obtained through Emergency Investigational New Drug application to the US Food and Drug Administration (*4*).

Diagnosis of Lassa fever in patients arriving from West Africa might be challenging for healthcare providers unfamiliar with the spectrum of its clinical presentation, a challenge that is also common to the consideration of other viral hemorrhagic fevers in returning travelers (*5*–*7*). Additionally, although Lassa virus is not transmitted through casual contact, contact-tracing investigations of returning case-patients have often been large in scale (*8*). To quantify the frequency of case-patients having distinctive clinical features, time from patient presentation to clinical suspicion of a Lassa fever diagnosis, and the risk for secondary Lassa virus transmission, we performed a retrospective review of all 33 reported cases of Lassa fever imported from West Africa during 1969–2016.

## Methods

We searched PubMed for publications using the terms “Lassa” and “Lassa fever.” We identified additional articles by reviewing references in retrieved reports (*9*) and official correspondence by public health officials involved in these cases. We selected 74 publications discussing clinical or epidemiologic aspects of the 33 imported Lassa fever cases for review and collected information pertaining to case demographics, distinctive clinical features suggestive of Lassa fever, time from patient seeking care to clinical suspicion of Lassa fever, and number of contacts traced. We defined distinctive clinical features as fever and >1 of the following: sore throat or pharyngitis, retrosternal chest pain, or proteinuria. We selected these features on the basis of the cumulative positive predictive value for fever, sore throat, retrosternal chest pain, and proteinuria for Lassa fever of 0.81 in a case–control study among 441 hospitalized patients in Sierra Leone (*10*). Although precise definitions varied between investigations, high-risk contacts were typically defined as contacts with substantial direct contact with patients or their body fluids.

## Findings

During 1969–2016, a total of 33 patients traveling from 7 West Africa countries to 9 other countries were diagnosed with Lassa fever (Appendix Table 1). The median age of these patients was 45 years (range 18–72 years). Potential sources of Lassa fever exposures varied. Eleven patients were healthcare workers working in West Africa with either known or suspected exposures to Lassa fever patients; 4 patients had known exposure to rodents or history of travel to rural areas in West Africa. The only known risk factor for 18 patients was living in or traveling to West Africa. Twenty patients had illness onset during the West Africa dry season (November–April), and 10 patients had onset during the wet season (May–October); time of year for disease onset was not specified for 3 patients.

Twenty patients traveled to their destination on a commercial airliner; of these, 12 were symptomatic during flight. Ten patients were medically evacuated, 6 of whom had a known or suspected exposure to Lassa fever at the time of evacuation. Information on method of travel was not available for 3 patients. At the time patients sought care, medical providers were aware of travel history to West Africa for 26 (87%) of 30 patients; ascertainment of travel histories by medical providers was not described for 3 cases.

Of the 29 patients for whom clinical information was available (Appendix Table 2), 17 (59%) had fever and >1 distinctive clinical features of Lassa fever. Time from patients seeking medical care to clinical suspicion of Lassa fever by clinical providers in their destination country ranged from 1 to 22 days (median 5 days). The time from when patients sought care to patient isolation ranged from 1 to 25 days (median 7 days). We found no reports of Lassa virus PCR testing performed on any patient before 2000; however, 9 of 16 patients (56%) in 2000 and later years had a positive Lassa virus PCR test within 1–2 days of hospital admission. Of the 32 patients for whom information on isolation procedures were described, 24 patients were isolated at some point during their hospitalizations in their destination countries. Of these, 11 (34%) patients were placed in a form of isolation immediately after they sought medical care; 3 patients were transferred to biocontainment units, and the remaining 8 patients were isolated with techniques ranging from standard precautions to a combination of contact, droplet, and airborne precautions. Of the 13 patients who were isolated later in their hospital stay, 2 patients were isolated with contact and airborne precautions, and 11 were subsequently transferred to specialized hospitals with infection control capacity designed for the care of patients with highly infectious diseases. Time to isolation ranged from 3 to 15 days after hospital admission (*11*). The last 2 patients who sought care in the United States were admitted to dedicated Ebola treatment units established during the 2014–2015 West Africa Ebola epidemic. Of the 31 patients for whom outcomes were described, 12 patients died, yielding a case-fatality rate of 39%.

Treatment regimens were described for 23 patients. Twelve (52%) patients initially received antimalarial medications or antimicrobial drugs because of clinical suspicion of malaria or another infectious disease during their treatment course. In total, intravenous ribavirin was ordered for 7 (30%) patients. Four patients received intravenous ribavirin; 2 received a full course, and the other 2 died during treatment. Three patients had intravenous ribavirin ordered but died before receiving the medication.

Contact tracing investigations were either not performed or not described in the literature for 16 (48%) patients. For the remaining 17 (52%) patients, a total of 3,420 contacts were followed; the number of contacts followed per investigation ranged from 3 to 552 (median 173). Eleven contact investigations stratified contacts into high-risk and low-risk contacts, with some further separating high-risk contacts into first-line or second-line contacts (*12*). High-risk contacts were defined as having substantial exposure to patients or their body fluids, such as through direct unprotected exposure to blood or other body fluids from a case-patient. By these criteria, 139 total contacts were defined as being high-risk across 11 investigations. In 9 investigations, high-risk contacts accounted for 2%–8% of total contacts; in 2 investigations, they accounted for 40%–60% of total contacts.

Only 2 cases of secondary transmission of Lassa virus occurred, both in Germany. Neither of the source case-patients for these 2 patients was isolated. The first instance of transmission occurred to a physician who performed a physical examination, obtained intravenous access, and obtained blood samples from a Lassa fever patient without wearing any personal protective equipment (*13*). Because of the physician’s high-risk exposure, ribavirin prophylaxis was initiated and completed. Serologic testing was performed and yielded IgG titers of 1:320 specific to the strain of Lassa virus from the case-patient, indicating probable seroconversion in the physician. However, the physician remained asymptomatic.

The second instance of secondary transmission, reported in 2016, occurred in a mortician who handled the body of a healthcare worker who was evacuated from Togo to Germany and diagnosed with Lassa fever retrospectively. The mortician reported wearing 2 pairs of gloves when handling the corpse but did not wear an apron or a facial mask. The mortician reported mild upper respiratory tract symptoms before contact with the deceased patient. However, 4 days after handling the corpse, his symptoms worsened. Six days after handling the corpse, the mortician tested positive for Lassa virus by real-time reverse transcription PCR. The mortician’s clinical course was notable for fever, upper respiratory tract symptoms, and pharyngeal erythema with exudates, myalgias, and arthralgias. He received intravenous ribavirin for 10 days and oral favipiravir for 4 days, with gradual resolution of his symptoms and clinical recovery (*14*,*15*). Contacts of this secondary case-patient were followed but did not indicate any evidence of further transmission.

## Discussion

The 33 cases of imported Lassa fever that occurred during 1969–2016 posed a similar set of challenges: timely diagnosis of a rare infectious disease not endemic to the patient’s destination country, timely treatment, and prevention of Lassa virus transmission to contacts. Among patients who were not medically evacuated, the median number of days from patient presentation to clinical suspicion of Lassa fever by clinicians in the destination country was 5 days. Several factors might have contributed to this delay in diagnosis. First, patients were seen by providers in countries where Lassa fever is not endemic, requiring consideration of a travel-associated illness infrequently encountered outside of West Africa. Second, in many cases, the patients’ travel to West Africa was not known at the time they initially sought care. Third, the clinical findings of Lassa fever are variable, ranging from nonspecific symptoms, such as fever, nausea, and myalgias in the early phase, to more distinctive features later, including pharyngitis, sore throat, tonsillitis, oropharyngeal ulcers, facial and neck swelling, conjunctival injection, and proteinuria. Hemorrhage is usually seen only in a minority of cases. Although fever and >1 distinctive clinical features can be suggestive of the diagnosis, they were only present in 59% of patients. In addition, of patients with a known travel history to West Africa, 12 (48%) did not demonstrate distinctive clinical features of Lassa fever. As such, providers encountering patients who have a nonspecific febrile illness after travel to West Africa should elicit a travel history and consider Lassa fever early in the differential diagnosis. Suspicion should be especially high for those patients with fever and >1 of the distinctive features we have described. Although most returning travelers from West Africa with Lassa fever in 2000 or later had viremia confirmed through a positive Lassa virus test obtained within 1–2 days of admission, some patients did not have their illness diagnosed until weeks into their illness. Samples of patients with suspected Lassa fever should be obtained as early as possible and tested by Lassa virus PCR at a reference laboratory; most reference laboratories in Europe and elsewhere have demonstrated proficiency in performing Lassa virus molecular diagnostics (*16*,*17*).

Treatment of Lassa fever comprises effective supportive care and use of intravenous ribavirin. Although timely treatment with intravenous ribavirin depends on successful procurement of the drug, it also rests on early consideration of the diagnosis, and might even be administered before laboratory confirmation of Lassa fever diagnosis in patients with severe illness. The relative minority of case-patients who received intravenous ribavirin in our review highlights the importance of early consideration of Lassa fever in the differential diagnosis for appropriate patients.

Infection control was another challenge encountered by medical providers and healthcare systems caring for Lassa fever patients. The lack of appropriate use of isolation or barrier precautions in the 2 instances of secondary transmission speaks to the importance of adhering to standard precautions when caring for all patients, regardless of their diagnosis or presumed infectious status. In addition, the case of secondary transmission to the mortician in Germany illustrates the importance of maintenance of standard precautions during autopsy. Early consideration of Lassa fever as a diagnosis might also enable early institution of isolation and prevention of secondary transmission. Among those case-patients for whom a specific form of isolation was specified, most were admitted to high-security containment facilities or negative-pressure rooms with airborne precautions. Although these forms of isolation can prevent secondary transmission of Lassa virus, simple barrier or contact precautions have also been demonstrated to be safe and are less expensive and labor-intensive (*5*,*18*).

Contact tracing investigations frequently involved hundreds of contacts and a substantial investment of time and labor on the part of public health teams. One investigation noted that “active surveillance of contacts by public health teams was impracticable and required enormous resources, involving over 3,000 communications” (*6*). Most investigations were similarly comprehensive, involving identification and longitudinal follow-up of case-patients’ friends, family, and casual contacts, including airplane passengers, as well as numerous healthcare staff. Contacts were often separated into 2 categories: high-risk (i.e., having substantial exposure to case-patients) and low-risk (i.e., having only casual contact or proximity to case-patients). However, body temperature monitoring, home visits, and serologic testing were frequently coordinated for contacts in both high- and low-risk categories. To minimize the burden on public health systems and maximize the likelihood of successful secondary case identification, future responses should consider focusing on investigating high-risk contacts exclusively.

Our review had several limitations. Information on historic cases, particularly those before 1985, was incomplete and limited. In some cases, reports provided scant or no information on the physical examination or laboratory studies of patients upon admission. Reports on contact tracing provided different degrees of detail, and levels of risk assessment were variable between investigations.

With the ease and frequency of international travel, Lassa fever will continue to be encountered by healthcare providers in countries where Lassa fever is not endemic. Strict maintenance of standard infection control precautions in healthcare is critical for all patients and will help prevent secondary transmission of Lassa virus. Timely recognition of distinctive clinical features, earlier treatment of patients, and targeted public health responses focused on high-risk contacts will also be important components of future responses to imported cases of Lassa fever.

Appendix Characteristics of imported Lassa fever cases, 1969–2016.
