# Supplementary material for: Lassa Fever in Travelers from West Africa, 1969–2016
Source: Emerg Infect Dis. 2019 Feb;25(2):236–9. doi: 10.3201/eid2502.180836 (PMC6346466; doi:10.3201/eid2502.180836)
Supplement: Appendix — Characteristics of imported Lassa fever cases, 1969–2016. [file 18-0836-Techapp-s1.pdf]

Article DOI: https://doi.org/10.3201/eid2502.180836

Lassa Fever in Travelers from West Africa, 1969–2016

Appendix

Appendix Table 1. Demographic and travel-related characteristics of imported Lassa fever (LF) cases, 1969–2016

Case no.	Year	Origin country	Destination country	Age, y	Sex	Suspected route of exposure
1 (*1–5*)	1969	Nigeria	United States (NY)	52	F	Nurse in Nigeria caring for LF patients
2 (*6*)	1971	Sierra Leone	United Kingdom	?	F	Nurse in Nigeria caring for LF patients
3 (*6*)	1971	Sierra Leone	United Kingdom	?	M	Doctor in Sierra Leone likely caring for LF patients
4 (*7*)	1972	Sierra Leone	United Kingdom	35	F	Nurse in Sierra Leone, needle-stick from patient with unknown illness who later died
5 (*8–10*)	1974	Nigeria	Germany	?	M	Doctor in Nigeria caring for physician with LF
6 (*11,12*)	1975	Nigeria	United Kingdom	39	M	Doctor in Nigeria
7 (*13–17*)	1976	Sierra Leone	United States (DC)	42	F	Lived in Sierra Leone in house with rodents
8 (*18*)	1976	Nigeria	United Kingdom	33	M	Working in Nigeria
9 (*19–22*)	1980	Burkina Faso	Netherlands	34	M	Working in Burkina Faso, manure seen near house
10 (*23–25*)	1981	Nigeria	United Kingdom	18	F	Lived/worked in Nigeria
11 (*26*)	1982	Nigeria	United Kingdom	21	F	Lived in Nigeria
12 (*27,28*)	1984	Sierra Leone	United Kingdom	47	M	Rodent exposure while camping in Sierra Leone
13 (*29–31*)	1985	Sierra Leone	United Kingdom	27	F	Midwife in Sierra Leone caring for LF patients
14 (*32*)	1987	Sierra Leone, Liberia	Israel	47	M	Travel to rural Sierra Leone and Liberia
15 (*33*)	1987	Sierra Leone	Japan	48	M	Worked in Sierra Leone
16 (*34*)	1989	Nigeria	Canada	38	M	Worked in Nigeria in agriculture
17 (*35,36*)	1989	Nigeria	United States (IL)	43	M	Attended funeral of relative in Nigeria who died from febrile illness diagnosed as malaria
18 (*37–41*)	2000	Cote d'Ivoire, Burkina Faso, Ghana	Germany	22	F	Travel to Cote d'Ivoire, Burkina Faso, Ghana
19 (*37,42–45*)	2000	Sierra Leone	United Kingdom	50	M	Aid worker in eastern Sierra Leone
20 (*46–49*)	2000	Nigeria	Germany	56	M	Unknown
21 (*50–55*)	2000	Sierra Leone	Netherlands	48	M	Surgeon working at hospital with LF patients
22 (*56*)	2003	Sierra Leone	United Kingdom	?	?	Work as soldier in rural Sierra Leone
23 (*57*)	2004	Sierra Leone, Liberia	United States (NJ)	38	M	Travel to farms in Sierra Leone and Liberia
24 (*58,59*)	2006	Sierra Leone	Germany	68	M	Travel to Sierra Leone
25 (*60*)	2007	Nigeria	South Africa	46	?	Public health physician from Nigeria
26 (*61*)	2009	Nigeria	United Kingdom	66	M	Travel to Nigeria
27 (*62*)	2009	Mali	United Kingdom	20s	M	Worked in rural Mali
28 (*63*)	2010	Liberia	United States (PA)	47	M	Travel to Liberia, sleeping in dwelling infested by living and dead rats
29 (*64,65*)	2014	Liberia	United States (MN)	46	M	Travel to Liberia
30 (*66–68*)	2015	Liberia	United States (NJ)	55	M	Work in Liberia, contact with rodents/excreta
31 (*69*)	2016	Togo	Germany	40s	M	Nurse in Togo
32 (*70*)	2016	Togo	United States (GA)	33	M	Nurse in Togo, cared for patient with LF
33 (*71,72*)	2016	Liberia	Sweden	72	F	Travel to rural Liberia, possible exposure to rodent excreta and acquaintances with LF

Appendix Table 2. Clinical and epidemiologic characteristics of imported Lassa fever cases, 1969–2016*

Case no.	Year	Initial clinical symptoms	Physical exam/basic lab findings	Positive LASV PCR test within 1-2 days of admission (N/A = not available)	Treatment (s)	Outcome	High-risk contacts/total contacts (% high-risk/total)†	Secondary cases
1 (*1–5*)	1969	Fever, sore throat, malaise, headache, nausea	Oropharyngeal ulcer, epigastric tenderness, lymphadenopathy, tremor, nystagmus, dizziness, scalp hair loss, muscle tenderness; leukopenia (neutrophil-predominant), anemia, elevated ESR, proteinuria	N/A	Hydroxychloroquine, procaine and crystalline penicillin, supportive treatment	Survived	Unknown	0
2 (*6*)	1971	Fever, iridiocyclitis, nausea/vomiting, anorexia	Abdominal tenderness	N/A	Chloramphenicol, ampicillin, hydrocortisone, prednisone, cloxacillin	Survived	Unknown	0
3 (*6*)	1971	Fever, malaise, anorexia, headache, joint pains, pleuritic and shoulder pain	Leukocytosis; mild thrombocytopenia, elevated ESR	N/A	None	Survived	Unknown	0
4 (*7*)	1972	Fever, headache, prostration, nausea/vomiting, limb and back pain	Hypotension; anemia, elevated ESR, microscopic hematuria	N/A	Chloroquine	Survived	Unknown	0
5 (*8–10*)	1974	Fever, malaise, pharyngitis, nausea/vomiting, subconjunctival hemorrhage, myalgias	Pharyngitis, soft palate ulceration, cervical adenopathy	N/A	Chloroquine, ampicillin, chloramphenicol, convalescent serum	Survived	3	0
6 (*11,12*)	1975	Fever, joint pains	Unknown	N/A	Unknown	Died	361	0
7 (*13–17*)	1976	Headache, vomiting, diarrhea, neck/back pain, headache, vertigo	Leukopenia	N/A	Unknown	Survived	29/552 (5%)	0
8 (*18*)	1976	Unknown	Unknown	N/A	Unknown	Survived	300	0
9 (*19–22*)	1980	Abdominal pressure, poor appetite, fever, rash, face and feet swelling	Conjunctival erythema, skin peeling at fingertips; elevated ESR	N/A	Chloroquine	Survived	Unknown	0
10 (*23–25*)	1981	Fever, abdominal pain, bilious vomiting, retro-orbital pain, facial edema, urinary frequency	Abdominal tenderness; leukocytosis, hyponatremia, microscopic hematuria and proteinuria, transaminitis, thrombocytopenia, coagulation abnormalities	N/A	Chloramphenicol, convalescent serum, supportive treatment	Survived	5/173 (3%)	0
11 (*26*)	1982	Fever, headache, fatigue	Leukocytosis, elevated bilirubin	N/A	Chloroquine, quinine, sulfadoxine/pyrimethamine	Survived	Unknown	0
12 (*27,28*)	1984	Fever	Unknown	N/A	Unknown	Survived	Unknown	0
13 (*29–31*)	1985	Fever, diarrhea, exudative pharyngitis,	Unknown	N/A	Chloroquine, quinine, chloramphenicol, oral ribavirin,	Survived	20/50 (40%)	0

Case no.	Year	Initial clinical symptoms	Physical exam/basic lab findings	Positive LASV PCR test within 1-2 days of admission (N/A = not available)	Treatment (s)	Outcome	High-risk contacts/total contacts (% high-risk/total)†	Secondary cases
		conjunctivitis, generalized tender lymphadenopathy			IV ribavirin, prostacyclin analogue, plasma, dexamethasone, mannitol			
14 (*32*)	1987	Fever, headaches	Exudative pharyngitis, hypotension, neurological signs/myelitis; leukopenia, thrombocytopenia, transaminitis	N/A	Supportive treatment	Survived	Unknown	0
15 (*33*)	1987	Fever, sore throat, malaise, diarrhea, epigastric pain	Facial edema, pharyngitis, axillary lymphadenopathy, papular rash on neck and chest, distended abdomen with ascites, hepatomegaly; transaminitis, proteinuria, ketonuria, elevated LDH, CPK, ESR, CRP	N/A	Supportive treatment	Survived	Unknown	0
16 (*34*)	1989	Fever, headache, malaise, nausea, chills, sore throat, dry cough, pleuritic chest pain	Inflamed conjunctiva and pharynx; leukopenia, albuminuria, elevated AST	N/A	Chloroquine, sulfadoxine/pyrimethamine, trimethoprim/sulfamethoxazole, chloramphenicol	Survived	Unknown	0
17 (*35,36*)	1989	Fever, shaking chills, sore throat, myalgia, persistent severe headaches	Transaminitis	N/A	Penicillin VK, cefaclor; ribavirin requested but not received prior to patient dying	Died	7/102 (7%)	0
18 (*37–41*)	2000	Fever, flu-like symptoms, tonsillitis	Pharyngitis, ulcerative tonsillitis; transaminitis, renal failure, thrombocytopenia, elevated LDH	Yes	Artesunate, ciprofloxacin, IV ribavirin	Died	Unknown	0
19 (*37,42–45*)	2000	Fever, malaise, diarrhea	Unknown	Yes	Unknown	Died	125	0
20 (*46–49*)	2000	Fever, diarrhea, one episode of generalized seizures	Transaminitis, elevated CSF protein, decreased glucose	Yes	Unknown	Died	18/232 (8%)	1
21 (*50–55*)	2000	Fever, malaise, nausea, diarrhea, myalgias, arthalgias	Rash on trunk; thrombocytosis, transaminitis, renal injury	No (diagnosis considered on day 6, PCR returned positive day 8)	Artesunate, cefmandol, netilmicin, doxycycline, IV ribavirin	Died	132	0
22 (*56*)	2003	Unknown	Unknown	Unknown	Unknown	Unknown	Unknown	0
23 (*57*)	2004	Fever, chills, severe sore throat, diarrhea, back pain	Unknown	No	Antimalarial, antibiotic therapy; IV ribavirin requested but not received prior to patient dying	Died	5/188 (3%)	0
24 (*58,59*)	2006	Fever, worsening of pre-existing neurological symptoms	Unknown	No (first sample was sent on day 10 and tested positive)	Unknown	Unknown	Unknown	0
25 (*60*)	2007	Unknown	Unknown	Yes	Unknown	Died	Unknown	0
26 (*61*)	2009	Fever, malaise, loss of appetite, abdominal	Unknown	Yes	IV ribavirin requested but not received prior to patient dying	Died	0/328 (0%)	0

Case no.	Year	Initial clinical symptoms	Physical exam/basic lab findings	Positive LASV PCR test within 1-2 days of admission (N/A = not available)	Treatment (s)	Outcome	High-risk contacts/total contacts (% high-risk/total)†	Secondary cases
		pain, confusion, lethargy, mild diarrhea						
27 (*62*)	2009	Unknown	Unknown	Yes	Antimalarial therapy	Died	7/125 (6%)	0
28 (*63*)	2010	Fever, chills, knee/ankle pain, anorexia, sore throat, skin tenderness, shortness of breath	Parotid enlargement, tonsillar exudates, posterior cervical lymphadenopathy, splenomegaly; leukopenia, thrombocytopenia, transaminitis	No (diagnosis considered on day 3, first sample was sent on day 5 and tested positive)	None	Survived	0/140 (0%)	0
29 (*64,65*)	2014	Fever, nausea, vomiting, diarrhea	Confusion, generalized abdominal pain, proteinuria	Yes	Dialysis, methylprednisolone	Survived	6/255	0
30 (*66–68*)	2015	Fever, chills, myalgias, sore throat	Pharyngeal erythema and exudates, tender cervical lymphadenopathy; transaminitis, renal injury	Yes	Broad-spectrum antibiotics; IV ribavirin requested but not received prior to patient dying	Died	15/177 (8%)	0
31 (*69*)	2016	Fever, malaise, sore throat	Unknown	No (diagnosed postmortem)	Anti-malarials, broad-spectrum antibiotics	Died	33/55 (60%)	1
32 (*70*)	2016	Fevers, sore throat, retro-orbital headache, diminished hearing, diarrhea, malaise, weakness	Conjunctival pallor, oral thrush, systolic murmur, bladder distention with suprapubic tenderness; leukopenia, thrombocytopenia, renal injury, transaminitis	Yes	IV ribavirin, oral favipiravir	Survived	Unknown	0
33 (*71,72*)	2016	Fever, nausea, arthralgia, loose stools, headache	Atrial fibrillation; elevated CRP, renal injury, transaminitis, proteinuria	No (diagnosis considered on day 22, first sample was sent on day 24 of sample from day 15 which tested positive)	None	Survived	122	0

*AST, aspartate aminotransferase; IV, intravenous; CPK, creatine phosphokinase; CRP, C-reactive protein ; CSF, cerebrospinal fluid; ESR, eryhthrocyte sedimentation rate; LASV, Lassa virus LDH, lactate dehydrogenase; N/A, not available.
†For those investigations that did not specify number of high-risk contacts, number refers to total contacts.

References

1. Frame JD, Baldwin JM Jr, Gocke DJ, Troup JM. Lassa fever, a new virus disease of man from West Africa. I. Clinical description and pathological findings. Am J Trop Med Hyg. 1970;19:670–6. PubMed http://dx.doi.org/10.4269/ajtmh.1970.19.670

2. Leifer E, Gocke DJ, Bourne H. Lassa fever, a new virus disease of man from West Africa. II. Report of a laboratory-acquired infection treated with plasma from a person recently recovered from the disease. Am J Trop Med Hyg. 1970;19:677–9. PubMed http://dx.doi.org/10.4269/ajtmh.1970.19.677

3. Buckley SM, Casals J. Lassa fever, a new virus disease of man from West Africa. 3. Isolation and characterization of the virus. Am J Trop Med Hyg. 1970;19:680–91. PubMed http://dx.doi.org/10.4269/ajtmh.1970.19.680

4. Speir RW, Wood O, Liebhaber H, Buckley SM. Lassa fever, a new virus disease of man from West Africa. IV. Electron microscopy of Vero cell cultures infected with Lassa virus. Am J Trop Med Hyg. 1970;19:692–4. PubMed http://dx.doi.org/10.4269/ajtmh.1970.19.692

5. Casals J, Buckley S, Frame JD, Leifer E. Isolation and characterization of "Lassa" virus—Connecticut and New York. MMWR Morb Mortal Wkly Rep. 1969;18:293–4.

6. Gilles HM, Kent JC. Lassa fever: retrospective diagnosis of two patients seen in Great Britain in 1971. BMJ. 1976;2:1173. PubMed http://dx.doi.org/10.1136/bmj.2.6045.1173

7. Woodruff AW, Monath TP, Mahmoud AAF, Pain AK, Morris CA. Lassa fever in Britain: an imported case. BMJ. 1973;3:616–7. PubMed http://dx.doi.org/10.1136/bmj.3.5881.616

8. Corey L. Suspect outbreak of Lassa fever in Onitsha, Nigeria. Unpublished investigation report; 1974.

9. Reinhardt. Outbreak suspect Lassa fever. Unpublished investigation report; 1974.

10. Hutchins D. Evaluation of Lassa fever. Unpublished investigation report; 1974.

11. Vella EE. Lassa fever. Trans R Soc Trop Med Hyg. 1975;69:430–1.

12. Lassa fever. Wkly Epidemiol Rec. 1975;3.

13. Zweighaft RM, Fraser DW, Hattwick MAW, Winkler WG, Jordan WC, Alter M, et al. Lassa fever: response to an imported case. N Engl J Med. 1977;297:803–7. PubMed http://dx.doi.org/10.1056/NEJM197710132971504

14. Sheagrin JN, Oner VO, Pate JR, Wolfe M. Possible Lassa fever—Washington, D.C. MMWR Morb Mortal Wkly Rep. 1976;25:64.

15. Pate JR. Follow-up on Lassa fever—Washington, D.C. MMWR Morb Mortal Wkly Rep. 1976;25:68.

16. Wolfe M, Oner VO, Pate JR. Follow-up on Lassa fever—Washington, D.C. MMWR Morb Mortal Wkly Rep. 1976;25:83.

17. Lassa fever. Wkly Epidemiol Rec. 1976;15:119.

18. Suspect case of Lassa fever. Wkly Epidemiol Rec. 1976;33:264.

19. Clayton AJ. Lassa fever, Marburg and Ebola virus diseases and other exotic diseases: is there a risk to Canada? Can Med Assoc J. 1979;120:146–55. PubMed

20. Van der Heide RM. Een patient met Lassakoorts uit Opper-Volta, herkend in Nederland. Ned Tijdschr Geneeskd. 1982;314:20–2.

21. Unexpected diagnosis of Lassa fever. Commun Dis Rep CDR Wkly. 1980;80:4.

22. Lassa fever surveillance. Wkly Epidemiol Rec. 1981;6:48.

23. Cooper CB, Gransden WR, Webster M, King M, O'Mahony M, Young S, et al. A case of Lassa fever: experience at St Thomas's Hospital. Br Med J (Clin Res Ed). 1982;285:1003–5. PubMed http://dx.doi.org/10.1136/bmj.285.6347.1003

24. Emond RT, Bannister B, Lloyd G, Southee TJ, Bowen ETW. A case of Lassa fever: clinical and virological findings. Br Med J (Clin Res Ed). 1982;285:1001–2. PubMed http://dx.doi.org/10.1136/bmj.285.6347.1001

25. Banatvala JE. A case of Lassa fever. Br Med J (Clin Res Ed). 1982;285:1653. PubMed http://dx.doi.org/10.1136/bmj.285.6355.1653

26. Bowen ETW, Emond RTD. A case of Lassa fever. Commun Dis Rep CDR Wkly. 1982;82:3.

27. Emond RTD, Weir WRC, Bowen ETW, Lloyd G, Southee T. Managing Lassa fever. Lancet. 1984;2:926. PubMed http://dx.doi.org/10.1016/S0140-6736(84)90679-2

28. Lassa fever: Ex Sierra Leone. Commun Dis Rep CDR Wkly. 1984;84:1.

29. Fisher-Hoch SP, Price ME, Craven RB, Price FM, Forthall DN, Sasso DR, et al. Safe intensive-care management of a severe case of Lassa fever with simple barrier nursing techniques. Lancet. 1985;2:1227–9. PubMed http://dx.doi.org/10.1016/S0140-6736(85)90752-4

30. Glover SC, Fisher-Hoch SP. Management of Lassa fever. Lancet. 1985;2:1359. PubMed http://dx.doi.org/10.1016/S0140-6736(85)92651-0

31. "Mission Midwife." Midwives Chron Nurs Notes. 1389(98):138.

32. Schlaeffer F, Bar-Lavie Y, Sikuler E, Alkan M, Keynan A. Evidence against high contagiousness of Lassa fever. Trans R Soc Trop Med Hyg. 1988;82:311. PubMed http://dx.doi.org/10.1016/0035-9203(88)90458-0

33. Hirabayashi Y, Oka S, Goto H, Shimada K, Kurata T, Fisher-Hoch SP, et al. An imported case of Lassa fever with late appearance of polyserositis. J Infect Dis. 1988;158:872–5. PubMed http://dx.doi.org/10.1093/infdis/158.4.872

34. Mahdy MS, Chiang W, McLaughlin B, Derksen K, Truxton BH, Neg K. Lassa fever: the first confirmed case imported into Canada. Can Dis Wkly Rep. 1989;15:193–8. PubMed

35. Holmes GP, McCormick JB, Trock SC, Chase RA, Lewis SM, Mason CA, et al. Lassa fever in the United States. Investigation of a case and new guidelines for management. N Engl J Med. 1990;323:1120–3. PubMed http://dx.doi.org/10.1056/NEJM199010183231607

36. Conrad JL, Mahy BWJ. Investigation of a Confirmed Lassa Case. Unpublished investigation report; 1989.

37. Haas WH, Breuer T, Pfaff G, Schmitz H, Köhler P, Asper M, et al. Imported Lassa fever in Germany: surveillance and management of contact persons. Clin Infect Dis. 2003;36:1254–8. PubMed http://dx.doi.org/10.1086/374853

38. World Health Organization. Imported case of Lassa fever in Germany—update [cited 2017 Aug 23]. http://www.who.int/csr/don/2000_01_18a

39. World Health Organization. Lassa fever in Germany [cited 2017 Aug 23]. http://www.who.int/csr/don/2000_01_13

40. Günther S, Emmerich P, Laue T, Kühle O, Asper M, Jung A, et al. Imported lassa fever in Germany: molecular characterization of a new lassa virus strain. Emerg Infect Dis. 2000;6:466–76. PubMed http://dx.doi.org/10.3201/eid0605.000504

41. Robert Koch Institute. Anmerkungen zu einem importierten Lassa-Fieber-Erkrankungsfall. Epid Bull. 2000;3:23–4.

42. Crowcroft NS, Meltzer M, Evans M, Shetty N, Maguire H, Bahl M, et al. The public health response to a case of Lassa fever in London in 2000. J Infect. 2004;48:221–8. PubMed http://dx.doi.org/10.1016/j.jinf.2003.11.009

43. World Health Organization. Suspected Lassa fever, UK [cited 2017 Aug 23]. http://www.who.int/csr/don/2000_03_13

44. World Health Organization. Suspected Lassa fever, UK—confirmation [cited 2017 Aug 23]. http://www.who.int/csr/don/2000_03_14

45. Jones J. Lassa fever imported to England. Euro Surveill. 2000;4.

46. Günther S, Weisner B, Roth A, Grewing T, Asper M, Drosten C, et al. Lassa fever encephalopathy: Lassa virus in cerebrospinal fluid but not in serum. J Infect Dis. 2001;184:345–9. PubMed http://dx.doi.org/10.1086/322033

47. Kiehl W, Haas W. A case of Lassa fever imported into Wiesbaden, Germany. Euro Surveill. 2000;4.

48. World Health Organization. Imported case of Lassa fever in Germany [cited 2017 Aug 23]. http://www.who.int/csr/don/2000_04_04

49. Fock R, Wirtz A, Finke E-J, Koch U, Scholz D, Niedrig M, et al. Management und Kontrolle lebensbedrohender hochkontagioser Infektionskrankheiten. Bundesgesundheitsblatt Gesundheitsforschung Gesundheitsschutz. 1999;5:389–401. http://dx.doi.org/10.1007/s001030050124

50. Swaan CM, van den Broek PJ, Wijnands S, van Steenbergen JE. Management of viral haemorrhagic fever in the Netherlands. Euro Surveill. 2002;7:48–50. PubMed http://dx.doi.org/10.2807/esm.07.03.00338-en

51. Wijnands S, van Steenbergen J. Public health management of fatal case of Lassa fever in the Netherlands. Euro Surveill. 2000;4.

52. World Health Organization. Imported case of Lassa fever in the Netherlands—update [cited 2017 Aug 23]. http://www.who.int/csr/don/2000_07_26

53. Swaan CM, van den Broek PJ, Kampert E, Berbée GAM, Schippers EF, Beersma MFC, et al. Management of a patient with Lassa fever to prevent transmission. J Hosp Infect. 2003;55:234–5. PubMed http://dx.doi.org/10.1016/j.jhin.2003.08.002

54. Schmitz H, Köhler B, Laue T, Drosten C, Veldkamp PJ, Günther S, et al. Monitoring of clinical and laboratory data in two cases of imported Lassa fever. Microbes Infect. 2002;4:43–50. PubMed http://dx.doi.org/10.1016/S1286-4579(01)01508-8

55. Imported case of Lassa fever in the Netherlands. Euro Surveill. 2000;4.

56. World Health Organization. Imported case of Lassa fever in United Kingdom [cited 2018 May 15]. http://www.who.int/csr/don/2003_02_10a

57. Aufiero P, Karabulut N, Rumowitz D, Shah S, Nsubuga J, Piepszak B, et al.; Centers for Disease Control and Prevention (CDC). Imported Lassa fever—New Jersey, 2004. MMWR Morb Mortal Wkly Rep. 2004;53:894–7. PubMed

58. E-alert 24 July: Case of Lassa fever imported into Germany from Sierra Leone. Euro Surveill. 2006;11:E060727.1. PubMed

59. World Health Organization. Imported case of Lassa fever in Germany [cited 2017 Aug 23]. http://www.who.int/csr/don/2006_07_25

60. Archer B, Blumberg L, Jansen van Vuren P, Kemp A, Leman P, Le Roux C, et al. Viral haemorrhagic fever outbreaks in South Africa, 2007–2009. NICD Commun Dis Surveill Bull. 2010;8:15–6.

61. Kitching A, Addiman S, Cathcart S, Bischop L, Krahé D, Nicholas M, et al. A fatal case of Lassa fever in London, January 2009. Euro Surveill. 2009;14:14. PubMed

62. Atkin S, Anaraki S, Gothard P, Walsh A, Brown D, Gopal R, et al. The first case of Lassa fever imported from Mali to the United Kingdom, February 2009. Euro Surveill. 2009;14:14. PubMed

63. Amorosa V, MacNeil A, McConnell R, Patel A, Dillon KE, Hamilton K, et al. Imported Lassa fever, Pennsylvania, USA, 2010. Emerg Infect Dis. 2010;16:1598–600. PubMed http://dx.doi.org/10.3201/eid1610.100774

64. Centers for Disease Control and Prevention. Lassa fever reported in U.S. traveler returning from West Africa [cited 2017 Aug 16]. https://www.cdc.gov/media/releases/2014/p0404-lassa-fever.html

65. Choi MJ, Worku S, Knust B, Vang A, Lynfield R, Mount MR, et al. A case of Lassa fever diagnosed at a community hospital—Minnesota 2014. Open Forum Infect Dis. 2018;5:ofy131. PubMed http://dx.doi.org/10.1093/ofid/ofy131

66. Kulkarni PA, Chew D, Youssef-Bessler M, Hamdi HA, Montoya LA, Cervantes KB, et al. Case report: imported case of Lassa fever—New Jersey, May 2015. Am J Trop Med Hyg. 2018;99:1062–5. PubMed http://dx.doi.org/10.4269/ajtmh.17-0316

67. World Health Organization. Lassa fever—United States of America [cited 2017 Aug 16]. http://www.who.int/csr/don/28-may-2015-lassa-fever-usa-en

68. Lehmann C, Kochanek M, Abdulla D, Becker S, Böll B, Bunte A, et al. Control measures following a case of imported Lassa fever from Togo, North Rhine Westphalia, Germany, 2016. Euro Surveill. 2017;22:22. PubMed http://dx.doi.org/10.2807/1560-7917.ES.2017.22.39.17-00088

69. World Health Organization. Lassa fever—Germany [cited 2017 Aug 16]. http://www.who.int/csr/don/23-march-2016-lassa-fever-germany/en

70. Raabe VN, Kann G, Ribner BS, Morales A, Varkey JB, Mehta AK, et al.; Emory Serious Communicable Diseases Unit. Favipiravir and Ribavirin Treatment of Epidemiologically Linked Cases of Lassa Fever. Clin Infect Dis. 2017;65:855–9. PubMed http://dx.doi.org/10.1093/cid/cix406

71. Grahn A, Bråve A, Lagging M, Dotevall L, Ekqvist D, Hammarström H, et al. Imported case of Lassa fever in Sweden with encephalopathy and sensorineural hearing deficit. Open Forum Infect Dis. 2016;3:ofw198. PubMed http://dx.doi.org/10.1093/ofid/ofw198

72. World Health Organization. Lassa fever—Sweden [cited 2017 Aug 23]. http://wwww.who.int/csr/don/8-april-2016-lassa-fever-sweden
